# Technological approaches to streamline vaccination schedules, progressing towards single-dose vaccines

**DOI:** 10.1038/s41541-020-00238-8

**Published:** 2020-09-18

**Authors:** Giuseppe Lofano, Corey P. Mallett, Sylvie Bertholet, Derek T. O’Hagan

**Affiliations:** GSK, Slaoui Center for Vaccines Research, Rockville, MD 20850 USA

**Keywords:** Vaccines, Drug development, Business strategy in drug development, Drug delivery, DNA vaccines

## Abstract

Vaccines represent the most successful medical intervention in history, with billions of lives saved. Although multiple doses of the same vaccine are typically required to reach an adequate level of protection, it would be advantageous to develop vaccines that induce protective immunity with fewer doses, ideally just one. Single-dose vaccines would be ideal to maximize vaccination coverage, help stakeholders to greatly reduce the costs associated with vaccination, and improve patient convenience. Here we describe past attempts to develop potent single dose vaccines and explore the reasons they failed. Then, we review key immunological mechanisms of the vaccine-specific immune responses, and how innovative technologies and approaches are guiding the preclinical and clinical development of potent single-dose vaccines. By modulating the spatio-temporal delivery of the vaccine components, by providing the appropriate stimuli to the innate immunity, and by designing better antigens, the new technologies and approaches leverage our current knowledge of the immune system and may synergize to enable the rational design of next-generation vaccination strategies. This review provides a rational perspective on the possible development of future single-dose vaccines.

## Introduction

In May 2015, Doctors Without Borders along with the South Sudan Ministry of Health and the National Cholera Taskforce had to face a tough decision. In Juba, South Sudan, a *Vibrio cholerae* outbreak had just been declared, but only 250,000 doses of the *Shanchol* vaccine were available for the over 500,000 citizens of Juba. Clearly there were not enough doses to fully protect the entire population with the recommended two-dose regimen. With minimal help from epidemiological evidence, the healthcare workers and government agreed to offer a single dose of the *Shanchol* vaccine to high-risk city areas in order to rapidly immunize as many persons as possible. After the outbreak, a vaccine effectiveness study showed that the single-dose intervention had 80.2% effectiveness (unadjusted, 95% CI 61.5–100.0), with a remarkable positive impact on public health of Juba^1^. This is an important lesson that highlights the need to focus on achieving maximal vaccine coverage during an outbreak. Reducing the number of doses administered to achieve the necessary levels of protection helped to save many lives in Juba and highlights the social and economic value of the potential of next-generation single-dose vaccines.

Vaccines are typically administered to children and require multi-dose series of injections to induce an adequate level of protection. Although the value of vaccination is unquestionable, UNICEF and WHO, that monitor the completion of the vaccine series worldwide, warn that we are still far from achieving a universal immunization coverage; for example, in 2018, at least 19.4 million children worldwide did not complete the recommended three-dose series of the Diphtheria, Tetanus, and Pertussis (DTP) vaccine^2^. The DTP vaccine is one of the most widely used vaccines in the world and it is considered a benchmark for comparing the quality of national healthcare systems in providing routine immunization services^2^. Almost 6 million children worldwide started but did not complete the DTP series suggesting that all those children could be fully protected if a single-dose vaccine course was available (https://www.who.int/immunization/monitoring_surveillance/data/en/). Strikingly, there are many vaccines for which children worldwide do not reach full compliance with the recommended schedules (https://www.cdc.gov/nchs/fastats/immunize.htm, https://www.who.int/immunization/policy/immunization_tables/en/)^3,4^, underlining the importance of renewed efforts toward the designing of single-dose vaccines. On top of that, children are typically naïve for any foreign antigen and may have suboptimal immune responses, all of which makes any vaccine design effort more challenging. How then to induce a strong priming and long-lasting immunity in children with a single-dose vaccine? Vaccinologists are currently exploring a number of technological solutions to this challenge.

Vaccines that require a lower number of doses are typically made with a live attenuated form of the target pathogen against which the vaccine will confer protection. For example, the MMR vaccine is a live attenuated vaccine against measles, mumps, and rubella, and confers protection in children after a single vaccination; a second dose is recommended only several years later (https://www.who.int/immunization/policy/immunization_tables/en/). Another example is represented by the live attenuated yellow fever vaccine: a single dose is enough to confer sustained protective immunity against yellow fever disease; a booster dose is not necessary (https://www.who.int/immunization/policy/immunization_tables/en/). Although those vaccines were once believed to confer lifelong immunity with just few doses, accumulating evidence suggest that a silent natural infection from the circulating pathogen may instead be required to acquire long-term protection^5^. Live attenuated vaccines can bring safety challenges to some segments of the population such as immunocompromised individuals, and sometimes they provide suboptimal efficacy for certain diseases, such as varicella^6^. In the past 30 years, the use of subunit antigens coupled with the advances in the recombinant DNA technology have enabled the development of a new generation of safe and effective vaccines. Subunit vaccines typically include two components: the antigen, a part of the pathogen against which to generate protective immunity, and the adjuvant, a substance that enhances the body’s immune response to the antigen. Subunit vaccines are considered safer but generally less effective than live attenuated vaccines and require multiple doses to induce protective immunity.

In this review we discuss the approaches that vaccinologists are considering for the design of next generation subunit vaccines and how to induce potent immune responses with fewer injections, ideally just one. Reducing the number of vaccine doses brings remarkable value to society but is not an easy task and poses a big challenge for clinicians and lab scientists. It has been at least 40 years that vaccinologists have been testing several approaches to design potent single-dose vaccines. So far, all the attempts have been unsuccessful, but have helped to reveal the limitations of past approaches and guide the development of new ones. Today we have a better understanding of the spatio-temporal dynamics of the immune responses after priming, all of which is being applied for the rational design of vaccines that ideally induce more potent immune responses after just one administration. Furthermore, the delivery of appropriate immune stimuli and the design of better antigens is also helping to come up with better vaccines. The immunological mechanisms of the vaccine-specific immune responses and the technologies to modulate those mechanisms are reviewed.

## Value of single-dose vaccines

In general, the design of single-administration vaccines aims to achieve three major objectives: (1) to improve the likelihood of worldwide coverage of vaccination, (2) to decrease the costs associated with multi-dose regimens, and (3) improve patient convenience. Incomplete immunization coverage is a major cause of preventable illness and death in both high-income and low-income countries (3,6). A study on vaccination coverage among US children under 2 years old has reported that only 30% receive all the six recommended vaccinations on time, indicating that too many children do not receive vaccines at age-appropriate times or never complete the recommended vaccine schedules^3,4^. Several socioeconomic factors have been associated with low country vaccination coverage including government health spending, difficulty to deliver the vaccines and educational variables^4,7,8^, indicating that additional country-specific policy, educational, and clinical interventions—not only technological innovation—are required to facilitate vaccine uptake. The complexities in achieving global vaccination coverage and immunization equity are many and have been discussed elsewhere^9–11^. Clearly, reducing the number of vaccine injections may maximize vaccination coverage and improve vaccine effectiveness as ease of vaccine uptake may sometimes help to compensate for vaccines with less-than-ideal efficacy^12,13^, especially for those pathogens for which a highly effective vaccine is difficult to design such as the influenza virus^14,15^. Single-administration vaccines might also help to decrease the costs associated with multi-dose regimens and improve patient convenience, as fewer injections and healthcare visits will be needed to provide immunity, especially in certain target populations that require quick and protective immune responses, including travelers, patients expecting imminent surgery, and during outbreaks.

Historically, a great approach to simplify immunization schedules and improve vaccine coverage has relied on the adoption of combination vaccines. Two of the most used combination vaccines are the Diphtheriae, Tetanus and acellular Pertussis (DTaP), a subunit vaccine, and the Measles, Mumps and Rubella (MMR) a live attenuated vaccine. Although combination vaccines have helped a lot in improving vaccine coverage and extending efficacy against the pathogens included in the vaccines^16^, not all vaccines can be combined into a single-injection. Indeed, there is always the risk that the efficacy (or safety) of the combination might be less than that seen with the administration of the vaccines separately^17,18^. A commonly reported example of immune interference occurs in the MMRV vaccine. The MMRV vaccine, a combination of MMR and Varicella vaccines, does result in fewer injections than if the two vaccines were administered separately, but it is also associated with higher risk of fever within 42 days after vaccination, when used as a first dose at ages 12–23 months^19,20^; indeed, the CDC advices that MMR vaccine and varicella vaccines might be administered as separate injections for the first dose in children 12–47 months of age. In another example, a reduction in Hib-specific antibody titers is typically reported for the hexavalent vaccine DTaP-HBV-IPV/Hib. The mechanisms of immune interference are not completely understood, but it seems that the Hib antigen is not compatible with alum, the adjuvant present in the vaccine^15,18,21^; nevertheless, the Hib compatibility problem seem to be solved with a new vaccine formulation^22^. In general, a combination of multiple antigens often results in big challenges for vaccine scale up and development.

Because of a combination of the above reasons, there is a clear value in the designing of vaccination strategies with fewer vaccine doses. But how to do that? A key approach aims at translating our current knowledge of the mechanisms of the immune responses into technological innovation for a rational design of potent single-dose vaccines. This approach so far has shown to be problematic, mostly because of the technical challenges that vaccinologists face when trying to elicit strong immune responses with few vaccine doses, all of which is discussed in the next sections.

## Clinical evidence for optimized vaccination schedules

Vaccination schedules are typically based on series of 3–4 doses within 4–6 months, with some vaccines requiring a booster dose through lifetime. Multi-dose series are used for at least two reasons: (1) to drive desired immune response in an individual to an adequate level of protection and (2) equally if not more important from a public health perspective, to increase the percentage of the vaccinated population that are no longer susceptible and achieve an “herd effect”. When trying to optimize the vaccination schedules, it is often difficult to determine whether fewer doses of the same vaccine would still confer protection, because for most vaccines the immune correlates are not well defined, making it difficult to predict the level of protection that would be reached with a vaccine schedule that is different from the one assessed during the clinical trials. Nonetheless, post-marketing epidemiology studies, meta-analysis and ad-hoc clinical trials comparing different schedules side-by-side, have provided important evidence that certain vaccines can be protective after fewer doses and some regulatory authorities have updated their recommended vaccine schedules accordingly. Here are a few examples. The schedule of the AS04-adjuvanted HPV vaccine (Cervarix from GSK), initially approved with a three-dose schedule, was updated with a two-dose schedule following several post-marketing studies in 9–25-year-old women in which the level of safety and immunogenicity was observed to be similar between the two vaccine regimens^23–26^. The adjuvant AS04, which has a composition of aluminum hydroxide and 3-O-desacyl4′-monophosphoryl lipid A (MPL-A), is believed to be responsible for the high level of vaccine-induced antibody titers, a surrogate of protection for HPV, and persist for several years in vaccinated women, even after a single vaccine dose^24–27^; Cervarix is currently recommended with a two-dose schedule as well as another HPV vaccine, Gardasil (Merck), which contains aluminum hydroxy phosphate sulfate as an adjuvant. Another example of an updated vaccination schedule is Prevnar 13 (Pfizer), an aluminum-adjuvanted pneumococcal vaccine, for which it has been shown that for 9 of the 13 serotypes included in the vaccine, the post-booster responses after a single priming are equivalent or superior to those seen following the standard 2 priming + 1 boost schedule, indicating that a schedule with fewer doses still confers protection^28^. Another recent example is represented by Heplisav-B, a CpG ODN-adjuvanted vaccine against Hepatitis B virus, which is administered with a two-dose schedule and shows similar safety and immunogenicity profile to three doses of other HBV vaccines^29^. A key commonality among all those vaccines is that they use well-optimized adjuvanted formulation, all of which highlights the importance of using vaccine adjuvants not only to modulate the vaccine-specific immune responses, but also to reduce the number of doses required to reach protection with subunit-based vaccines. Interestingly, Cervarix, Gardasil, and Heplisav-B also present a nanoparticle-based antigen structure, suggesting that a combination of multicopy antigen display and immune adjuvant formulations may provide key strategies for the rational design of streamlined vaccination schedules.

Several decades ago, when vaccinologists began to dissect the mechanism of action of the first successful vaccines, it became clear that the development of new technologies and approaches would have been essential to design vaccination strategies with fewer injections. Since then, extensive research has been generated, new technologies and approaches have been explored, but no potent single-dose vaccine has yet reached the clinic. In the next sections we discuss major historical accomplishments and challenges for vaccinologists, and we summarize the new technologies that are enabling the preclinical development of single-dose vaccines.

## History of attempts to develop single-dose vaccines

The concept of single-dose vaccines dates back nearly 40 years and received most attention with the WHO Special Program for Vaccine Development initiated in the 1980s^30^. The WHO initiative focused on the development of single-dose vaccines for the low-income countries and a key strategy involved the use of controlled release technologies for a slow release of vaccine antigens, with the scope of mimicking multi-dose regimes or natural infections. The primary target to employ the new release technologies was neonatal tetanus, at that time a major threat for public health because of the hurdles to reach full compliance with the multi-dose vaccine schedule required to confer protection^30,31^. The WHO initiative led to the evaluation of biodegradable polymer-based microparticles for the delivery of antigens including tetanus toxoid (TT), diphtheria toxoid, hepatitis B antigen and HIV envelope glycoprotein gp120^31^.

In the late 1970s Langer et al. first showed that a single vaccination with a polymer-based vaccine formulation could induce long-lasting antibody titers in animal models^32,33^. The polymer was bio-erodible and allowed for slow and sustained antigen release, whilst also providing an adjuvant effect. Subsequently, extensive research has focused on the development of single-dose vaccines, mostly using the established biodegradable polymer, poly(lactide-co-glycolide) (PLG) nanoparticle technologies for controlled antigen delivery. O’Hagan et al. showed long-term vaccine-specific antibody responses in mice following subcutaneous immunization with ovalbumin entrapped in PLG-based biodegradable nanoparticles^34^. In another study Men et al.^35^ prepared nanoparticles with entrapped TT that induced high TT-specific antibody and T cell responses in mice following a single immunization, with TT-specific IgG titers similar to those observed after three injections of TT adsorbed to alum^35^. Many other groups reported similar findings with antigens, such as diphtheria toxoid, hepatitis B antigen, and HIV envelope glycoprotein gp120 (reviewed by Siddhartha, J. et al. and O’Hagan, D.T. et al.^33,36^) demonstrating that controlled antigen release can provide a successful approach in small animal models.

Although preclinical testing provided promising results for the use of PLG nanoparticles, technical limitations in the manufacturability of such vaccines arose. Unfortunately, the process to prepare PLG particles typically damaged the stability of the antigen and impaired its immunogenicity^37,38^. In most groups, PLG microparticles were prepared through a process of antigen encapsulation that involved the use of organic solvents, agitation, and the creation of interfaces. Moreover, upon degradation in vivo, the polymer created a low pH environment, all of which led to protein unfolding and aggregation, with subsequent loss of functional epitopes and antigenicity^39–42^. Furthermore, clinical manufacturing of vaccines requires the product to be either produced under aseptic conditions or terminally sterilized, all of which may endanger antigen stability in PLG nanoparticles. Several research groups in the past decades have tried to address these manufacturability issues and have made many attempts to stabilize entrapped antigens, with limited success. As an alternative approach, Singh et al. successfully developed a way to obviate the need for encapsulation, by preparing the PLG particles separately and then adsorbing the antigen on the particles; while the surface adsorption of antigen does not allow for controlled release of antigen, such an approach does take advantage of the adjuvant properties of particulate antigen delivery^43–45^. In another study, Malyala et al. developed a two-stage process in which PLG microparticles were first sterilized by γ-irradiation, avoiding the need for aseptic manufacturing, and then incubated with reconstituted, sterile antigens to allow surface adsorption; the adsorbed PLG vaccines induced potent immune responses^46^. Most recently, Tzeng et al. showed a process for PLG encapsulation of an inactivated polio vaccine that involved the use of excipients to stabilize the formalin-fixed antigens and helped to preserve its stability^47^. Nevertheless, although this is an important advance which could contribute to the eventual eradication of polio, it should be appreciated that the complex formulation challenges required to stabilize a multicomponent combination vaccine in PLG microparticles still represent a potentially insurmountable problem. However, biodegradable PLG-based vaccines remain attractive and if the technology can be combined with other new approaches for the development of potent and efficient single-dose vaccines, promise remains.

## Better antigen design

The design of optimal vaccination strategies begins with the selection of the right antigen. In the past, vaccine antigens were typically selected through an empirical approach in which the pathogen (or a component of it) was isolated, inactivated and injected to induce protective immunity; many vaccines in use today have been developed with this empirical approach. In the last two decades a new and rational approach, named reverse vaccinology^48^, has enabled the development of new vaccines for which an empirical approach had not been successful, including a licensed vaccine against serogroup B meningococcus and many others are in clinical development^48,49^. By combining the most advanced technologies in genome sequencing, proteomics and bioinformatics, the reverse vaccinology approach has provided a framework to select the right vaccine antigens starting from the pathogen’s genome. Reverse vaccinology has pioneered new ways of thinking about vaccine development, including a new structural vaccinology approach, and represents a milestone in the history of vaccinology^50,51^.

Structure-based antigen design, also known as structural vaccinology, is today a key driving force in vaccine innovation^48,49,52^. It relies on bioinformatics and computational tools to design optimized protein antigens for an efficient display of protective epitopes to B cells. The structural vaccinology approach has been used in at least three new vaccine design strategies. One strategy aims to stabilize proteins or polypeptides in a protective antibody-inducing conformation, typically a conformation that is not very stable by itself, but it can be stabilized by modifying the sequence of the protein; this approach has led to the development of an RSV candidate vaccine and is helping in the development of the first universal influenza vaccine and HIV vaccine^53–55^. A second approach aims to merge multiple variants of the same protective epitope into a single chimeric protein or polypeptide chain; such approach has been successful to provide vaccine candidates including Factor H binding protein (FHbp) from serogroup B meningococcus and the backbone protein of pilus from serogroup B streptococcus^52,56^. A third approach aims to design self-assembling proteins (nanoparticles or virus-like particles) that display multiple copies of protective epitopes, which is a very efficient way to induce potent B cell responses^57–59^. Nanoparticles have been successfully used to develop HBV and HPV vaccines and can be used as scaffolds to design highly immunogenic multivariant antigens. For example, in the works from Yassine et al. and Kanekiyo et al., ferritin, a self-assembling protein nanoparticle with robust thermal and chemical stabilities, has been used as a scaffold to present a multivalent array of influenza virus hemagglutinin (HA) with its native trimeric conformation intact; this structure-based design strategy has led to the development of a potent and broad-coverage influenza vaccine that is now being tested in a phase I clinical trial^60–63^. In another study from Marcandalli et al. a single-immunization with a nanoparticle-based RSV vaccine outperformed the non-nanoparticle formulation in inducing T follicular helper (Tfh) cells and germinal center (GC) B cells^64^, highlighting the value of adopting a strategy of multi-copy antigen display in the designing of next-generation vaccines. More recently, a study by Tokatlian et al. has shown that nanoparticles induce potent GC responses in an immunogen glycan-dependent manner, suggesting that heavy glycosylated nanoparticles may have an advantage over the less glycosylated ones in promoting strong B cells responses^65^. Overall, a wide body of evidence indicates that nanoparticle-based antigens are more immunogenic than monovalent recombinant antigens. Nonetheless, it is unlikely that a single dose of a nanoparticle antigen would be adequate to induce robust and long-lasting immune responses in a clinical setting, especially in naïve subjects; however, a combination of all the strategies presented below may succeed^66–68^.

## Stimulating the innate immunity

Potent and long-lasting vaccine-specific adaptive immune responses occur only after adequate stimulation of the innate immunity. In the past century, during the early studies on the mechanisms of action of live attenuated vaccines, it became clear that a key aspect of the robust immunogenicity of these vaccines was their ability to stimulate innate immune cells through pattern recognition receptors (PRRs). PRRs are a series of germline-encoded host proteins, mostly expressed by innate immune cells, that may recognize pathogen-associated molecular patterns (PAMPs), which are molecules associated with microbial pathogens, or damage-associated molecular patterns (DAMPs), which are molecules associated with components of host’s cells that are released during cell damage or death. In the past 30 years, there has been a revolution in our understanding of the cells, receptors, and molecules that contribute to innate immunity and in the ways that the innate response directs the subsequent adaptive immune responses^69,70^, all of which has led to design immunological adjuvants to include in subunit vaccines and induce potent antigen-specific immune responses. Most adjuvants directly target PRRs, including TLR-agonists such as monophosphoryl lipid A (MPL-A), a component of AS04-adjuvanted and AS01-adjuvanted vaccines; other adjuvants, including MF59, AS03, and QS-21, activate tissue inflammation pathways with mechanisms that involve DAMPs^71,72^. We refer to other reviews for a comprehensive description of the mechanisms of action of adjuvants, but we would like to highlight that a key advantage offered by vaccine adjuvants is that they can be used not only to increase the quantity, but also to modulate the quality of the vaccine-specific immune response. For example, it is well known that the oil-in-water emulsions MF59 and AS03 may help to expand the antigen-specific antibody repertoire, whereas, for example, AS01 adjuvant may help to stimulate both humoral and cell-mediated immunity^73–78^. Although there are limited data on the immune responses measured after just a single dose of adjuvanted vaccines, it was shown that a single dose of an MF59-adjuvanted H5N1 vaccine was enough to induce a 3-fold increase in the frequency of virus-specific total CD4+ T cells which accurately predicted the rise of neutralizing antibodies against pandemic influenza, supporting the use of MF59 adjuvant in single-dose pre-pandemic influenza vaccines^79^. Adjuvants help to modulate the vaccine-specific immune response toward a desired immunological phenotype or a correlate of protection, and represent an outstanding tool for the designing of more potent single-dose vaccines^73,80,81^.

Viral vector-based vaccines mimic viral infections, display a constellation of immune evasion mechanisms, and activate PRRs that ultimately lead to potent immune activation^82–98^. As an example, most adenoviruses enter cells upon binding to the coxsackievirus and adenovirus receptor (CAR)^99^, and the viral double-stranded DNA is sensed by TLRs in the endosome and by NOD-like receptors in the cytoplasm leading to a pro-inflammatory cytokine response^50,52^. However, in some cases innate immunity is less important than antigen persistence in driving potent vector-induced immunity, as shown in the work of Quinn et al. in which adenoviral vaccine potency was independent of IFN and STING signaling^100^. Not only viral vectors, but also DNA and RNA vaccines can be sensed by PRRs on innate immune cells and bring an intrinsic adjuvant effect that contributes to the potent immune responses observed with these vaccines^101–103^.

## Modulating the release kinetics of the vaccine components

Two observations led scientists to speculate that antigen persistence after vaccination is an important factor for potent vaccine-specific immune responses. One observation came from studies on viral infections or live attenuated vaccines in which viral replication makes the antigen available for extended time and this is typically associated with strong antibody responses^104–108^. A second observation came from studies on the mechanisms of action of alum, the most widely deployed vaccine adjuvant, which was initially thought to deliver a slow release of the antigen, a mechanism called “depot” effect^71,109–111^. Although more recent studies have challenged this paradigm for alum mechanism of action, today it is widely accepted that prolonged antigen exposure leads to strong vaccine-specific immune responses^82,112–117^. Many recent works have provided further evidence: antigen exposure in draining lymph nodes over periods of at least several days induces an optimal cytokine profile^118^, enhances the differentiation of Tfh cells^119–121^, stimulates potent germinal center responses and improves the quality of the antibodies^114,120,122–124^. For example, a recent study from Cirelli et al., in which an HIV antigen was delivered with osmotic pumps over up to 4 weeks under the skin of non-human primates, shows that persistent antigen availability induces improved HIV-specific GC responses and antibody quality, probably with a mechanism that involves enhanced immune complex deposition on follicular dendritic cells^114,124^. All of this provides a better understanding of the immunological mechanisms after vaccination and raises key questions on how to translate preclinical concepts into clinical applications.

Multiple materials and technologies, each coming with specific biophysical properties and antigen release kinetics, are currently being explored in preclinical settings to enable a programmed antigen release after a single vaccination. How long antigen persistence should be, and if there is an optimal release kinetic, are still unclear points. Evidence suggests that antigen persistence should be maintained for more than 2–3 weeks in order to appreciate an improved immune response when compared to a single bolus injection: there seems to be a “lag” phase in which the presence of extra antigen does not make the immune responses stronger; this phase seems to last for around 14 days after the first antigen exposure, until the peak of the GC reactions^69,112,114^. Instead, 2–3 weeks of continuous antigen exposure not only is associated with stronger immune responses but also may help to preserve subdominant protective epitopes in the appropriate conformations^124^. The kinetic of antigen persistence also plays an important role: “exponential increase” outperforms a “nearly constant” release kinetic^112^. Although it is still difficult to design a vaccine that may allow for an exponentially increased delivery of the antigen in vivo, some materials, such as PLGA microparticles, can be programmed with a “pulsatile” release kinetic, in which the antigen is released in multiple sequential waves after a single vaccination, somehow mimicking a multi-bolus schedule. Alternatively, most of the technologies enable a “zero-order kinetic” in which a nearly constant release of the antigen persists for days or weeks. The most relevant materials and technologies that are being evaluated for the development of single dose vaccines are described below.

Although a variety of materials have been approved in humans for controlled release of small drugs and hormones^125,126^, none has been approved for controlled release of vaccines. Major obstacles that have limited the adoption of selected materials for vaccine development include: (a) difficulties in maintaining stability of the antigen (on-shelf and in vivo after injection); (b) challenges in controlling desired release kinetics; and (c) manufacturing constraints^36,82,127^. Nonetheless, a variety of materials and devices have been tested in preclinical studies and seem promising for clinical applications. PLG was the first biopolymer to be tested in the attempts to develop single-dose vaccines. As discussed above, although several technical limitations so far have prevented widespread clinical application of PLG-based vaccines, PLG microspheres have been widely published and clinically validated for the controlled release of proteins, peptides, and small molecules^36,82,126,128^, with the potential of co-delivering antigens and adjuvants^129–131^. A single-dose vaccine of stabilized PLG microparticles may allow for a pulsatile release of the antigen and the timing of the “bursts” can be potentially modulated by changing the biochemical and biophysical properties of the PLG microparticles^36,132^; however, a continuous release kinetic is also possible^133–135^. Other relevant materials that offer an alternative to the use of the PLG polymer are silica, dextran, collagen, chitosan, hydrogels, and fibroin from silk^82,136–139^; these materials are biodegradable, appear safe, can encapsulate a protein antigen and typically release it with zero-order release kinetics that can last for weeks after a single dose injection^82^. As examples, collagen minipellets can induce continuous antigen release for up to 14 days^140^, chitosan up to 35 days^134^. Although very few preclinical immunogenicity studies have been reported so far with these materials, they are in the toolkit for the development of future vaccine formulations for controlled antigen release.

Although the polymers described above are mostly used in the form of microparticles for intramuscular antigen delivery, they can also be engineered to design devices that deliver the antigens through the skin. The best example for this approach is represented by microneedle skin patches which encompass an array of solid pyramidal or cylindrical projections of few microns in size and are designed to mechanically perforate the stratum corneum to enter the epidermis and/or upper dermis upon application to the skin. Microneedles can be coated with dried vaccine formulations, or the microneedles themselves can comprise dissolving polymers that release the vaccine with programmable kinetics. Vaccine administration via microneedles provides several key advantages over traditional intramuscular injections by allowing for minimal pain and discomfort, by targeting the myriad of innate immune cells in the skin^141^, and by providing a way to encapsulate the bioactive molecules in a stable and lyophilized state prior to use^142^. Several types of microneedle patches that can tailor vaccine kinetics have been designed and tested in mice, NHPs, and early clinical trials, including microneedles composed of polymers that swell or dissolve at controlled rates when applied to the skin, releasing encapsulated vaccines^136,143–145^. For example, a type of dissolving microneedle patch made of carboxylmethylcellulose and trehalose was used to deliver cell-culture-derived influenza vaccine^146^; another type of patch used silk protein-based microneedles to release vaccines with extended kinetics in the skin, promoting T cell and humoral responses following vaccination^136^.

## Alternative approaches to promote persisting antigen availability

Other than biodegradable materials, at least three more approaches can be used to induce a programmable delivery of the antigen after vaccination; those approaches are based on the use of nucleic acids, viral vectors, or adjuvants. Although each of them has complex mechanisms of action, here we focus on reviewing their properties as antigen delivery systems.

For many years, DNA and RNA have been used in vaccination strategies, indeed nucleic acids can be efficiently engineered with the sequence of an antigen of interest and used to induce long-term in vivo expression of the antigen^101,102,147^. Mode of action studies of DNA-based and RNA-based vaccines have been extensively reviewed^102,103,148^ and are outside the scope of this review, however it is very important to note that both DNA-based and RNA-based vaccines potentially induce long-term antigen expression in vivo with kinetics that can resemble a zero-order antigen release, and may generate strong immune responses after a single dose; for example studies with luciferase-encoding DNA have shown that the luciferase can be detected for at least 3–4 weeks after a single vaccine injection^149–155^, suggesting that a DNA vaccine would be suitable to replace at least the second dose in a two-dose, 1-month apart vaccination schedule. Nevertheless, after its initial emergence in the 1990s, the level of interest in DNA as a vaccine modality gradually decreased as it became clear that the technology lacked potency when used in human subjects.

RNA vaccines are a more recent and more potent tool to induce persistent antigen expression and several works have been published in the recent years showing that one immunization of RNA vaccines may be sufficient to induce strong and protective immunity. For example, using a nucleoside-modified RNA platform, Pardi et al. have shown that a single dose RNA vaccine can protect from Zika virus infection^156^; in another work, by using a different platform with lipid-nanoparticle RNA vaccine, Bahl et al. have shown that an influenza vaccine was very immunogenic after just a single dose^157,158^; furthermore, recent clinical data have shown that a single vaccination with an mRNA-based vaccine boosted pre-existing serum neutralization titers against hMPV and PIV3, two significant causes of severe respiratory diseases for infants and children. Another very promising RNA vaccine platform is based on self-amplifying mRNA (SAM) vaccines which employ a modified version of the alphavirus genome and encode for the RNA replication machinery together with the gene of the antigen of interest^103^. SAM vaccines have several advantages over single-copy RNA vaccines, indeed SAM vaccines not only enable a large amount of antigen production from an extremely small dose of vaccine, but also allow for persistent antigen expression that seems to last for many weeks after a single vaccination^159,160^. SAM vaccines have been shown to provide strong B and T cell immunity against a variety of pathogens including influenza, HIV, RSV, Ebola, CMV, and Dengue^161^. Although RNA vaccines degrade faster than DNA vaccines, several modifications on the RNA structure and optimized delivery systems, including cationic nanoemulsions and lipid nanoparticles, can be used to improve RNA stability, in vivo sustained protein translation and the antigen-specific immune responses^102^.

Viral vectors represent the closest model to live attenuated vaccines, a benchmark for vaccine potency. Viral vectors enable potent stimulation of the innate immune system and persistent antigenic stimulation, all of which ultimately leads to potent humoral and cell-mediated immunity^82–90,100,162,163^. Factors that have limited the wide-spread use of viral vectors for clinical applications include: (a) the presence of pre-existing immunity against the viral vector which may limit its potency or give rise to unwanted side effects^164^; (b) evidence suggests that years of antigen stimulation may lead to exhaustion of the immune responses^165,166^, (c) replication-competent vectors may lead to chronic infections and pathogenesis^167^. In order to circumvent these potential limitations, several vectors have been developed for safe and effective use in humans including vectors based on replication-defective adenoviruses^82–90,168^, single-cycle adenoviruses^91–93^, vesiculoviruses^169^, herpes viruses^94^, or adeno-associated viruses (AAV)^95,170^. The most used class of viral vectors is represented by the replication-defective adenoviruses, small double-strand species-specific DNA viruses of human or non-human origin that are unable to establish chronic infections and may provide antigen persistence for months, depending on the animal model or viral strain considered^82–87,171^. For example, in a mouse model of immunization with a viral vector encoding for the SIV-Gag antigen, the number of antigen transcripts has been detected for at least 15 days after vaccination and most likely persist for longer^100^. In another study from Tatsis et al. using the AdC68 vector, antigen sequences were detected at the site of injection for at least 1 year after administration^83^. In both these studies a single immunization was sufficient to induce potent B and T cell responses, with a mechanism that seems independent of the vector-induced activation of innate immunity^100^. All of this evidence strongly supports the use of replication-defective adenoviruses as one of the best tools to induce strong immune responses with a single immunization. The replication-competent adeno-associated viruses (AAV) represent another class of extremely attractive vaccine carriers as they are highly versatile, they can readily be produced to high titers, they only encode the transgene product and are appear tolerated, even if given at very high doses^94,95^. AAVs have also been engineered as a gene therapy tool to deliver antibodies or other functional proteins, providing remarkable results in term of long-term gene expression. For example, AAVs have been shown to induce durable transgene expression for at least 12 months in mice^170–172^ and 4 months in rhesus macaques^173^. The only viral vector-based vaccines that has reached approval for clinical use so far is the rVSV-ZEBOV Ebola vaccine, which is based on the replication-competent vesicular stomatitis virus (VSV), a virus in the family Rhabdoviridae; the rVSV-ZEBOV vaccine has been recently used during the Ebola outbreak in the Democratic Republic of Congo in 2018, showing 97.5% vaccine effectiveness. We refer to a reference from Lauer et al. for a more detailed discussion on viral vectors used for clinical applications^174^.

A major potential weakness in using viral vectors as delivery systems is linked to the possibility of inducing immune cell exhaustion, a phenomenon sometime observed with chronic viral infections and in theory possible with any antigen persistence system. Immune cell exhaustion defines a state in which immune cells, typically T cells, following continuous and prolonged exposure to the antigen, become less responsive and exhausted^175,176^. T cell exhaustion usually manifests with several characteristic features, such as progressive and hierarchical loss of effector functions, sustained upregulation and co-expression of multiple inhibitory receptors, altered expression and use of key transcription factors, metabolic derangements, and homeostatic hypo-responsiveness^177^. Although it is not clear how long the exposure needs to occur before triggering an unfavorable exhausted immune phenotype, it is important that any vaccine strategy designed to induce persistent antigen exposure carefully evaluate and limit the occurrence of this phenomenon.

Vaccine adjuvants mediate immunostimulatory signals to the body’s immune system and promote potent vaccine-specific B and T cell responses. Although those signals are key for adjuvant mechanisms of action, we have evidence that certain adjuvants may form an antigen “depot” at the site of injection hereby promoting antigen slow release after vaccination. Initially a “depot effect” was hypothesized to explain the mechanism of action of alum-adjuvanted vaccines^71,178^; instead new findings of the past decades have suggested that an antigen depot might be far less important than alum-induced inflammasome activation in the modulation of alum adjuvanticity^71,109–111^. Importantly, a recent paper from Moyer et al. has shown that if the absorption of the antigen on alum is very strong, this may form antigen nanoparticles that slowly traffic to the lymph nodes—peaking up to 8 days after injection—and prolongs antigen bioavailability in vivo; this mechanism seems to be very important in shaping both the quantity and the quality of the antigen-specific immune responses of alum-adjuvanted vaccines^179^. A “depot effect” was also hypothesized to be important for the mechanisms of action of the oil-in-water emulsion MF59 adjuvant, but by using an immunization model with a labeled antigen, it was shown that the presence of MF59 did not significantly modify the distribution of the antigen^132^. Nonetheless, MF59 has been shown to induce antigen-specific GC B cells for at least 4 months after last immunization^180^, suggesting that antigen is retained in its native form for long-term GC activation all of which might contribute to the strong and broad B cell responses typically seen with MF59-aduvanted vaccines^71^. Adjuvants that may form an antigen depot at the site of injection also include the cationic adjuvant formulations (CAF platform) and the IC31 adjuvant. The principal component of the CAF adjuvant platform is the surfactant dioctadecylammonium (DDA) formulated into liposomes or emulsions, which modulate antigen biodistribution at the injection site^181^; Schmidt et al. have shown that intramuscular injection of a CAF-adjuvanted vaccine activates classical migratory dendritic cells that slowly transport the antigen to the draining lymph nodes and elicit strong T cell responses^182,183^. IC31 is another adjuvant that combines the immunostimulatory effects of an antibacterial peptide with the biochemical properties of a synthetic oligodeoxynucleotide (ODN1a, an agonist of the Toll-like receptor (TLR)-9) in order to induce potent antigen-specific immune responses^184^; Schellack et al. reported that an OVA peptide co-administered with IC31 adjuvant formed a depot at the injection site, which was still detectable 58 days after injection and was potentially responsible for the strong immune responses typically observed with IC31-adjuvanted vaccines^185^. CAF and IC31 adjuvants are currently in clinical trials for the development of a tuberculosis vaccine.

## Targeting site-specific delivery of the vaccine components

**Fig. 1:**
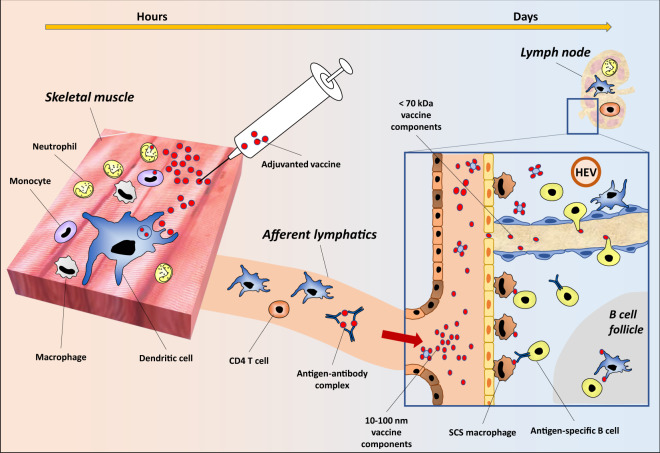
The journey of the vaccine components after injection. Intramuscular or subcutaneous injection triggers the recruitment of immune cells to the site of injection, where they activate, capture the antigen and migrate to the draining lymph node. Antigens, adjuvants or other components smaller than 10–100 nm may also diffuse in the lymphatic systems and reach the lymph node through the afferent lymphatics. While smaller molecules (<70 kDa) may diffuse through fenestrae of the subcapsular sinus, the largest molecules are transferred to the B cells with the help of subcapsular sinus macrophages. B cells and resident dendritic cells may also sense molecules in the conduits and transfer them to the B cell area to initiate the germinal center responses.

Although having a programmable system of antigen delivery is helpful, it might not be enough to efficiently stimulate all the players of the vaccine-specific immune responses. Indeed, appropriate stimuli—including the antigen—need to be displayed not only at the right time, but also in the right place. In general, there are two compartments that are particularly important in the context of vaccination: the site of injection, typically the muscle, and its draining lymph node (Fig. 1). A potent single-dose vaccine is required to induce the appropriate stimuli in each of those two sites.

To begin with, the skeletal muscle is typically where the vaccine first enters in the body: a resting muscle usually contains few immune cells, but vaccine administration triggers the recruitment of tissue resident and infiltrating immune cells, including professional antigen-presenting cells (APCs), which activate after vaccine administration^186^. With regard to vaccines platforms that are based on DNA or RNA delivery systems, they employ the expression machinery of the muscle cells in order to express the antigen at the injection site, which promotes the recruitment of immune cells^102^. With regard to subunit vaccines, antigens are typically delivered with vaccine adjuvants, that help to induce a transient inflammation in the muscle and further promote immune cell recruitment and activation^187^. The persistence and the quality of the adjuvant-induced immune stimuli, including cytokines and chemokines, represents a fingerprint of each adjuvant and provide qualitatively unique immune responses at the injection site. For example mouse studies have shown that frequencies of neutrophils and monocytes peak, respectively, at 16 and 48 h in MF59-injected muscles, while the AS01 adjuvant induces faster kinetics as both neutrophil and monocyte numbers peak already at 6 h^188–191^; adjuvant mechanisms of action are a complex and broad topic, and it will be briefly discussed below. Another important injection site is the skin; indeed, some vaccines are administered subcutaneously where they encounter a myriad of skin-resident APCs. For example, skin-resident dendritic cells and MHCII + Langerhans cells reside in different skin tissue layers and exhibit distinct time frames of lymph node homing^192^. Many materials for targeting skin dendritic cells have been explored, including hydrogels^143,193^ and large particulates^194^, with the common aim of localizing the materials to the site of administration to increase the likelihood of APC uptake and migration to the draining lymph node. Overall, the site of injection—skin or muscle—is a dynamic environment, where single-dose technologies may play an important role in modulating the “when” and the “how” antigens and adjuvants are released in order to trigger an efficient transport of the antigen from the injection site to the draining lymph node, where the adaptive response occurs.

Antigens and adjuvants may reach the draining lymph node in two different ways: through diffusion in the lymphatic vessels, or through cell-mediated transport. The lymphatic capillaries possess a fenestrated endothelium that allow for the passage of molecules 10–100 nm in diameter, including antigens and immune complexes, that flow through the lymphatic systems until they reach the subcapsular sinus (SCS) area of the lymph nodes^195,196^. Only molecules that are smaller than 70 kDa will be able to pass through the SCS and directly access to the B cell follicles, while bigger molecules will require an active transport from specialized cells, such as the poorly degradative SCS macrophages which capture immune complexes and other big molecules to mediate their transport to the follicles^197–200^. The different hydrodynamic properties of the lymphatic systems have been used in the past to design delivery systems that release molecules in specific sites of interest in the lymph node. For example, in a study by Lutz et al. adjuvant-loaded particles were encapsulated in pH-degradable hydrogel: the authors show that the hydrogel degraded over time and slowly released 50 nm nanoparticles that diffused away from the injection site and targeted dendritic cells in the draining lymph node hence inducing stronger immune responses with a mechanism that did not require cell-mediated transport^201^. In other studies, it has been shown that follicular B cells have also direct access to small soluble antigens^202^, even when those antigens are initially administered in the form of large nanoparticles^203^, suggesting that lymph proteases may release small antigens from large carriers, resulting in a free diffusion of the small molecules and direct access to B cells in the lymph nodes. For larger molecules, the story is different as they require active transport by immune cells. For example, immune complexes—vaccine antigens trapped by serum antigen-specific antibodies—are too big to get free access to the B cell follicles, instead they are transported to the lymph node by migratory immune cells, or diffuse in the subcapsular sinus (SCS) area until they are captured by SCS macrophages that transfer them to the B cells area^204^; typically SCS macrophages also capture heavy glycosylated antigens^205^, nanoparticles^206^, and liposomes. Importantly, liposomes are commonly used carriers owing to their amphipathic composition, which promotes internalization by endocytosis rather than scavenging by phagocytosis^207^. Once internalized, liposomes are processed by phospholipases, which disrupt their structure, causing the intracellular release of encapsulated cargo, including adjuvants and other small molecules^208^, and can be used for targeted delivery to SCS macrophages. Regarding the largest molecules, their transport from the injection site to the draining lymph node is modulated by the active transport of migratory immune cells. For example, dendritic cells and MHCII + Langerhans cells capture the antigen, migrate and localize in discrete draining lymph node locations^192^ to exert specific immunomodulatory functions^209^. Monocytes, neutrophils, myeloid dendritic cells, or plasmacytoid dendritic cells can also be selectively recruited by different vaccine adjuvants and can transport the antigen to the draining lymph node^210^. Finally, monoclonal antibody-coated microparticles can be engineered to deliver a cargo directly to the high endothelial venules of lymph nodes and modulate the immune responses^211^.

Overall those studies highlight the importance of targeted in vivo delivery of the vaccine components, which display unique hydrodynamic and immunological properties. In designing next generation vaccines, we need to take into account the many possible journeys of the vaccine components in vivo, indeed an optimal vaccine formulation may be specifically designed to target different cell types, in different places and even at different times, in order to induce potent immune responses with a single vaccination.

## Single-dose vaccines for the Coronavirus pandemic

The rapid expansion of the COVID-19 pandemic has made the development of a SARS-CoV-2 vaccine a global health and economic priority. Given the rapid evolution of the pandemic—hence for the need to quickly make available millions of doses of a vaccine to as many as possible and to rapidly generate protective immunity—a single-dose vaccination strategy is considered a hallmark of the target vaccine profile. At the time we write many vaccines are being evaluated in preclinical and clinical settings, some of which have already been shown to be safe and induce functional antibody titers after just a single vaccination. Those vaccines are designed to elicit protective immune responses against the S protein of SARS-CoV-2 and use RNA or viral vectors as antigen delivery systems. At least one RNA vaccine has reached phase III clinical testing, and encouraging data from a phase I trial have been published^212^. Although a two-doses schedule seems to be required to reach the desirable functional antibody titers, just one dose of the RNA vaccine was safe and enough to induce seroconversion in all study participants—with functional antibody titers in some of them—proving the promise of the RNA platform in providing some benefit as a single-dose strategy during pandemics. Adenoviral vectors are also showing very promising results as single-dose vaccine platform. In a phase I/II trial, a single dose of a chimpanzee adenovirus-vectored vaccine (ChAdOx1 nCoV-19) expressing the SARS-CoV-2 spike protein has been shown to induce neutralizing antibody responses against SARS-CoV-2 in 90–100% (depending on the test used to measure neutralizing titers) of vaccinated subjects^213^. In another phase II clinical trial a single vaccination with the non-replicating adenovirus type-5 (Ad5)-vectored COVID-19 vaccine has also been shown to induce significant neutralizing titers in the majority of the study participants^214^. Most recently, Mercado et al. have shown that a single dose of the adenovirus serotype 26 (Ad26) vector-based vaccine elicits neutralizing antibodies that correlate with protective efficacy in a non-human primate model of Coronavirus infection^215^. Clearly, all the potential of some of the technologies discussed in this review is being exploited in the fast-track and much-needed development of a single-dose Coronavirus vaccine.

## Conclusions

**Fig. 2:**
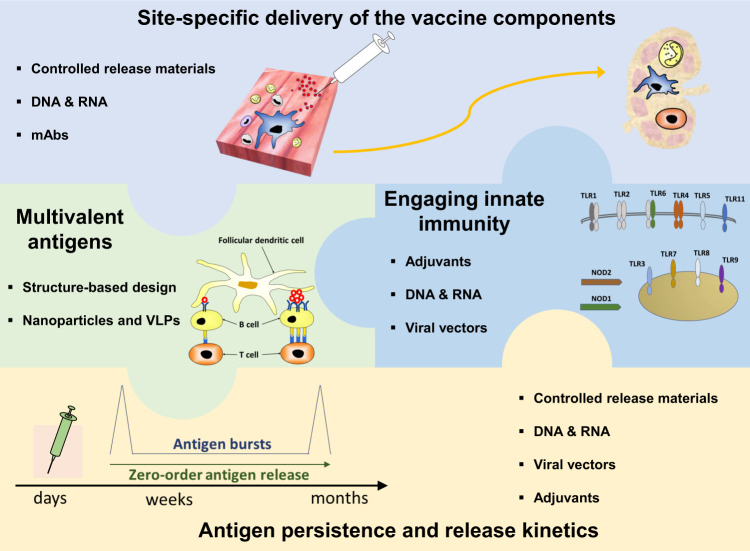
Approaches for the rational design of potent single-dose vaccines. A rational design of potent single-dose vaccines can be achieved by modulating the spatial and temporal deliveries of the vaccine components, by designing multivalent antigens and by efficiently engaging the innate immunity. The muscle and the draining lymph node are the two most important sites for targeted delivery of antigen and immune stimuli, which can be achieved by modulating the biophysical and biochemical properties of the vaccine components. Antigen persistence in germinal center regions of lymph nodes is essential to promote robust immune responses and can be modulated with controlled-release materials, DNA, RNA, viral vectors, or adjuvants. Antigens can be designed as virus-like particles (VLPs) to present a multivalent conformation and promote efficient B cell receptor engagement on the surface of B cells. Furthermore, activation of innate immune cells is essential to induce strong antigen-specific immune responses and can be achieved by engaging pattern recognition receptors with adjuvants, nucleic acids, and viral vectors vaccines.

Single-dose vaccines are in high demand by all stakeholders including patients, insurers, and vaccine companies, and vaccinologists may possess the right toolbox to begin to build them. Although early attempts in the development of single dose vaccines highlighted important technical challenges, today we have a better understanding of how the immune system works and we may have new tools to rationally design vaccines that induce strong immunity with fewer doses, ideally just one. Through the modulation of key immunological mechanisms, it might be possible to improve the quality and the quantity of vaccine-specific immune responses (Fig. 2). Several materials and technologies can be used to modulate the spatio-temporal availability of antigen and adjuvants in vivo; furthermore, the delivery of appropriate innate immune stimuli together with structure-optimized antigens are also very important for the rational design of more potent single-dose vaccines.

At least three important limitations in the development of single-dose vaccines still remain. First, many of the technologies described here have still to be tested in humans and, depending on their indications, their ability to work as single dose will have to be demonstrated in children, a key target population for the development of new vaccines. Second, the manufacturability for some of the technologies is still a major barrier for widespread clinical application; as examples, a manufacturing process for PLG-based vaccines has not yet been established. Third, any new vaccination strategy, regardless of the number of immunizations, needs to induce specific correlate(s) of protection, which are still unknown for many pathogens. Nonetheless, the approaches presented above can help to modulate not only the quantity but also the quality of vaccine-induced immune responses, providing a unique set of strategies to defeat vaccine-preventable diseases with a reduced number of vaccinations.

## References

[1] Azman AS (2016). Effectiveness of one dose of oral cholera vaccine in response to an outbreak: a case-cohort study. Lancet Glob. Health.

[2] Peck, M. et al. CDC. Global Routine Vaccination Coverage (2018). 10.15585/mmwr.mm6842a1 PMC6812836 31647786

[3] Kurosky SK, Davis KL, Krishnarajah G (2016). Completion and compliance of childhood vaccinations in the United States. Vaccine.

[4] Ventola, C. L. Immunization in the United States: recommendations, barriers, and measures to improve compliance. *P&T* (2016). PMC4927017 27408519

[5] Cohen, J. How long do vaccines last? The surprising answers may help protect people longer. https://www.sciencemag.org (2019).

[6] Chaves, S. S. et al. Loss of vaccine-induced immunity to Varicella over time. *New Engl. J. Med.* (2007). 10.1056/NEJMoa064040 17360990

[7] de Figueiredo A (2016). Forecasted trends in vaccination coverage and correlations with socioeconomic factors: a global time-series analysis over 30 years. Lancet Glob. Health.

[8] Gallagher KE (2016). Factors influencing completion of multi-dose vaccine schedules in adolescents: a systematic review. BMC Public Health.

[9] Lim SS, Stein DB, Charrow A, Murray CJL (2008). Tracking progress towards universal childhood immunisation and the impact of global initiatives: a systematic analysis of three-dose diphtheria, tetanus, and pertussis immunisation coverage. Lancet.

[10] Sodha SV, Dietz V (2015). Strengthening routine immunization systems to improve global vaccination coverage. Br. Med. Bull..

[11] Jacob V (2016). Increasing coverage of appropriate vaccinations: a community guide systematic economic review. Am. J. Prev. Med..

[12] Sah P, Medlock J, Fitzpatrick MC, Singer BH, Galvani AP (2018). Optimizing the impact of low-efficacy influenza vaccines. Proc. Natl Acad. Sci. USA.

[13] Fine P, Eames K, Heymann DL (2011). “Herd immunity”: a rough guide. Clin. Infect. Dis..

[14] Wei CJ (2020). Next-generation influenza vaccines: opportunities and challenges. Nat. Rev. Drug Discov..

[15] Poland GA (2018). Influenza vaccine failure: failure to protect or failure to understand?. Expert Rev. Vaccines.

[16] Kalies H (2006). The use of combination vaccines has improved timeliness of vaccination in children. Pediatr. Infect. Dis. J..

[17] Kurosky SK, Davis KL, Krishnarajah G (2017). Effect of combination vaccines on completion and compliance of childhood vaccinations in the United States. Hum. Vaccin Immunother..

[18] David AG, Skibinski BCB, Derek TO’Hagan (2010). Combination vaccines. J. Glob. Infect. Dis. 2011 Jan.-Mar..

[19] Jacobsen SJ (2009). Observational safety study of febrile convulsion following first dose MMRV vaccination in a managed care setting. Vaccine.

[20] Klein NP (2010). Measles–mumps–rubella–varicella combination vaccine and the risk of febrile seizures. Pediatrics.

[21] Eskola J (1999). Combined vaccination of *Haemophilus influenzae* type b conjugate and diphtheria–tetanus–pertussis containing acellular pertussis. Lancet.

[22] Xu J, Stek JE, Ziani E, Liu GF, Lee AW (2019). Integrated safety profile of a new approved, fully liquid DTaP5-HB-IPV-Hib vaccine. Pediatr. Infect. Dis. J..

[23] Romanowski B (2011). Immunogenicity and safety of the HPV-16/18 AS04-adjuvanted vaccine administered as a 2-dose schedule compared with the licensed 3-dose schedule: results from a randomized study. Hum. Vaccin.

[24] Romanowski B (2014). Immune response to the HPV-16/18 AS04-adjuvanted vaccine administered as a 2-dose or 3-dose schedule up to 4 years after vaccination: results from a randomized study. Hum. Vaccin Immunother..

[25] Kreimer AR (2011). Proof-of-principle evaluation of the efficacy of fewer than three doses of a bivalent HPV16/18 vaccine. J. Natl Cancer Inst..

[26] Markowitz LE, Drolet M, Perez N, Jit M, Brisson M (2018). Human papillomavirus vaccine effectiveness by number of doses: systematic review of data from national immunization programs. Vaccine.

[27] Van Damme P (2014). Effects of varying antigens and adjuvant systems on the immunogenicity and safety of investigational tetravalent human oncogenic papillomavirus vaccines: results from two randomized trials. Vaccine.

[28] Goldblatt D (2018). Pneumococcal conjugate vaccine 13 delivered as one primary and one booster dose (1+1) compared with two primary doses and a booster (2+1) in UK infants: a multicentre, parallel group randomised controlled trial. Lancet Infect. Dis..

[29] Hyer R, McGuire DK, Xing B, Jackson S, Janssen R (2018). Safety of a two-dose investigational hepatitis B vaccine, HBsAg-1018, using a toll-like receptor 9 agonist adjuvant in adults. Vaccine.

[30] Johansen P, Men Y, Merkle HP, Gander B (2000). Revisiting PLA/PLGA microspheres: an analysis of their potential in parenteral vaccination. Eur. J. Pharm. Biopharm..

[31] O'Hagan DT, Singh M, Gupta RK (1997). Poly(lactide-co-glycolide) microparticles for the development of single-dose controlled-release vaccines. Adv. Drug Deliv. Rev..

[32] Folkman, J. & Langer, R. Polymers for the sustained release of proteins and other macromolecules. *Nature* (1976). 10.1038/263797a0 995197

[33] Langer, R. S. & Preis, I. A single-step immunization by sustained antigen release. *J. lmmunol. Methods* (1979). 10.1016/0022-1759(79)90341-7 469267

[34] O’Hagan, D. T., Jeffery, H. & Davis, S. S. Long-term antibody responses in mice following subcutaneous immunization with ovalbumin entrapped in biodegradable microparticles. *Vaccine* (1993). 10.1016/0264-410x(93)90387-d 8212845

[35] Men Y, Thomasin C, Merkle HP, Gander B, Corradin G (1995). A single administration of tetanus toxoid in biodegradable microspheres elicits T cell and antibody responses similar or lsuperior to those obtained with aluminum hydroxide. Vaccine.

[36] Siddhartha J, O’Hagan DT, Singh M (2011). The long-term potential of biodegradable poly(lactide-co-glycolide) microparticles as the next-generation vaccine adjuvant. Expert Rev. Vaccines.

[37] Tacket CO (1994). Enteral immunization and challenge of volunteers given enterotoxigenic *E. coli* CFA/II encapsulated in biodegradable microspheres. Vaccine.

[38] Crotts G, Park TG (1998). Protein delivery from poly(lactic-co-glycolic acid) biodegradable microspheres: release kinetics and stability issues. J. Microencapsul..

[39] Griebenow CPAK (2001). Improved activity and stability of lysozyme at the water/CH2 Cl2 interface: enzyme unfolding and aggregation and its prevention by polyols. J. Pharm. Pharmacol..

[40] Zhu G, Mallery SR, Schwendeman SP (1999). Stabilization of proteins encapsulated in injectable poly (lactide-co-glycolide). Nat. Biotechnol..

[41] Ding AG, Schwendeman SP (2008). Acidic microclimate pH distribution in PLGA microspheres monitored by confocal laser scanning microscopy. Pharm. Res..

[42] Johansen P, Tamber H, Merkle HP, Gander B (1999). Diphtheria and tetanus toxoid microencapsulation into conventional and end-group alkylated PLA/PLGAs. Eur. J. Pharm. Biopharm..

[43] Singh M, Kazzaz J, Ugozzoli M, Malyala P, Chesko J, O’Hagan DT (2006). Polylactide-co-glycolide microparticles with surface adsorbed antigens as vaccine delivery systems. Curr. Drug Deliv..

[44] Singh M (2004). Anionic microparticles are a potent delivery system for recombinant antigens from *Neisseria meningitidis* serotype B. J. Pharm. Sci..

[45] Singh M (2004). Adsorption of a novel recombinant glycoprotein from HIV (Env gp120dV2 SF162) to anionic PLG microparticles retains the structural integrity of the protein, whereas encapsulation in PLG microparticles does not. Pharm. Res..

[46] Jain S (2011). A two-stage strategy for sterilization of poly(lactide-co-glycolide) particles by γ-irradiation does not impair their potency for vaccine delivery. J. Pharm. Sci..

[47] Tzeng SY (2018). Stabilized single-injection inactivated polio vaccine elicits a strong neutralizing immune response. Proc. Natl Acad. Sci. USA.

[48] Kulp DW, Schief WR (2013). Advances in structure-based vaccine design. Curr. Opin. Virol..

[49] Donnarumma D, Faleri A, Costantino P, Rappuoli R, Norais N (2016). The role of structural proteomics in vaccine development: recent advances and future prospects. Expert Rev. Proteom..

[50] Rappuoli R, Bottomley MJ, D’Oro U, Finco O, De Gregorio E (2016). Reverse vaccinology 2.0: human immunology instructs vaccine antigen design. J. Exp. Med..

[51] Sette A, Rappuoli R (2010). Reverse vaccinology: developing vaccines in the era of genomics. Immunity.

[52] Dormitzer PR, Grandi G, Rappuoli R (2012). Structural vaccinology starts to deliver. Nat. Rev. Microbiol..

[53] Joyce MG (2016). Iterative structure-based improvement of a fusion-glycoprotein vaccine against RSV. Nat. Struct. Mol. Biol..

[54] Kwong PD, Mascola JR (2018). HIV-1 vaccines based on antibody identification, B cell ontogeny, and epitope structure. Immunity.

[55] McLellan JS (2013). Structure-based design of a fusion glycoprotein vaccine for respiratory syncytial virus. Science.

[56] Nuccitelli A (2011). Structure-based approach to rationally design a chimeric protein for an effective vaccine against Group B *Streptococcus* infections. PNAS.

[57] Plummer EM, Manchester M (2011). Viral nanoparticles and virus-like particles: platforms for contemporary vaccine design. Wiley Interdiscip. Rev. Nanomed. Nanobiotechnol..

[58] Bachmann MF, Jennings GT (2010). Vaccine delivery: a matter of size, geometry, kinetics and molecular patterns. Nat. Rev. Immunol..

[59] Lopez-Sagaseta J, Malito E, Rappuoli R, Bottomley MJ (2016). Self-assembling protein nanoparticles in the design of vaccines. Comput. Struct. Biotechnol. J..

[60] Yassine HM (2015). Hemagglutinin-stem nanoparticles generate heterosubtypic influenza protection. Nat. Med..

[61] Kanekiyo M (2013). Self-assembling influenza nanoparticle vaccines elicit broadly neutralizing H1N1 antibodies. Nature.

[62] Kanekiyo M (2019). Mosaic nanoparticle display of diverse influenza virus hemagglutinins elicits broad B cell responses. Nat. Immunol..

[63] Swanson, K. A. et al. A respiratory syncytial virus (RSV) F protein nanoparticle vaccine focuses antibody responses to a conserved neutralization domain. *Sci. Immunol.* (2020). 10.1126/sciimmunol.aba6466 32358170

[64] Marcandalli J (2019). Induction of potent neutralizing antibody responses by a designed protein nanoparticle vaccine for respiratory syncytial virus. Cell.

[65] Tokatlian, T. et al. Innate immune recognition of glycans targets HIV nanoparticle immunogens to germinal centers. *Science* (2019). 10.1126/science.aat9120 PMC6420719 30573546

[66] Sankaranarayanan R (2018). Can a single dose of human papillomavirus (HPV) vaccine prevent cervical cancer? Early findings from an Indian study. Vaccine.

[67] Schiller JT, Lowy DR (2015). Raising expectations for subunit vaccine. J. Infect. Dis..

[68] Melo M (2019). Immunogenicity of RNA replicons encoding HIV Env immunogens designed for self-assembly into nanoparticles. Mol. Ther..

[69] Lofano G (2015). Oil-in-water emulsion MF59 increases germinal center B cell differentiation and persistence in response to vaccination. J. Immunol..

[70] Iwasaki A, Medzhitov R (2010). Regulation of adaptive immunity by the innate immune system. Science.

[71] De Gregorio E, D’Oro U, Wack A (2009). Immunology of TLR-independent vaccine adjuvants. Curr. Opin. Immunol..

[72] Coffman RL, Sher A, Seder RA (2010). Vaccine adjuvants: putting innate immunity to work. Immunity.

[73] Reed SG, Orr MT, Fox CB (2013). Key roles of adjuvants in modern vaccines. Nat. Med..

[74] Khurana, S. et al. Vaccines with MF59 adjuvant expand the antibody repertoire to target protective sites of pandemic avian H5N1 influenza virus. *Sci. Transl. Med.* **2**, (2010). 10.1126/scitranslmed.3000624 20371470

[75] Khurana, S. et al. MF59 adjuvant enhances diversity and affinity of antibody-mediated immune response to pandemic influenza vaccines. *Sci. Transl. Med.* **3**, (2011). 10.1126/scitranslmed.3002336 PMC3501657 21632986

[76] Khurana S (2014). Heterologous prime-boost vaccination with MF59-adjuvanted H5 vaccines promotes antibody affinity maturation towards the hemagglutinin HA1 domain and broad H5N1 cross-clade neutralization. PLoS ONE.

[77] Khurana, S. et al. AS03-adjuvanted H5N1 vaccine promotes antibody diversity and affinity maturation, NAI titers, cross-clade H5N1 neutralization, but not H1N1 cross-subtype neutralization. *NPJ Vaccines* **3**, 40. 10.1038/s41541-018-0076-2 (2018). 10.1038/s41541-018-0076-2 PMC6167326 30302282

[78] Coccia, M. et al. Cellular and molecular synergy in AS01-adjuvanted vaccines results in an early IFNgamma response promoting vaccine immunogenicity. *NPJ Vaccines* **2**, 25. 10.1038/s41541-017-0027-3 (2017). 10.1038/s41541-017-0027-3 PMC5627273 29263880

[79] Burny W (2017). Different adjuvants induce common innate pathways that are associated with enhanced adaptive responses against a model antigen in humans. Front. Immunol..

[80] Galli G (2009). Adjuvanted H5N1 vaccine induces early CD4+ T cell response that predicts long-term persistence of protective antibody levels. PNAS.

[81] De Gregorio E, Caproni E, Ulmer JB (2013). Vaccine adjuvants: mode of action. Front. Immunol..

[82] McHugh KJ, Guarecuco R, Langer R, Jaklenec A (2015). Single-injection vaccines: progress, challenges, and opportunities. J. Control Release.

[83] Tatsis N (2007). Adenoviral vectors persist in vivo and maintain activated CD8+ T cells: implications for their use as vaccines. Blood.

[84] Kim TS, Hufford MM, Sun J, Fu YX, Braciale TJ (2010). Antigen persistence and the control of local T cell memory by migrant respiratory dendritic cells after acute virus infection. J. Exp. Med..

[85] Finn JD (2009). Persistence of transgene expression influences CD8+ T-cell expansion and maintenance following immunization with recombinant adenovirus. J. Virol..

[86] Bassett JD, Swift SL, Bramson JL (2011). Optimizing vaccine-induced CD8(+) T-cell immunity: focus on recombinant adenovirus vectors. Expert Rev. Vaccines.

[87] Dicks MD (2015). The relative magnitude of transgene-specific adaptive immune responses induced by human and chimpanzee adenovirus vectors differs between laboratory animals and a target species. Vaccine.

[88] Tatsis N (2006). Chimpanzee-origin adenovirus vectors as vaccine carriers. Gene Ther..

[89] Lin J (2009). A new genetic vaccine platform based on an adeno-associated virus isolated from a rhesus macaque. J. Virol..

[90] Capone S (2013). Development of chimpanzee adenoviruses as vaccine vectors: challenges and successes emerging from clinical trials. Expert Rev. Vaccines.

[91] Peng B (2005). Replicating rather than nonreplicating adenovirus-human immunodeficiency virus recombinant vaccines are better at eliciting potent cellular immunity and priming high-titer antibodies. J. Virol..

[92] Crosby CM, Nehete P, Sastry KJ, Barry MA (2015). Amplified and persistent immune responses generated by single-cycle replicating adenovirus vaccines. J. Virol..

[93] Crosby CM, Barry MA (2014). IIIa deleted adenovirus as a single-cycle genome replicating vector. Virology.

[94] Lin A, Balazs AB (2018). Adeno-associated virus gene delivery of broadly neutralizing antibodies as prevention and therapy against HIV-1. Retrovirology.

[95] Nieto K, Salvetti A (2014). AAV vectors vaccines against infectious diseases. Front. Immunol..

[96] Del Giudice G, Rappuoli R, Didierlaurent AM (2018). Correlates of adjuvanticity: a review on adjuvants in licensed vaccines. Semin Immunol..

[97] Roelvink, P. W. et al. The coxsackievirus-adenovirus receptor protein can function as a cellular attachment protein for adenovirus serotypes from subgroups A, C, D, E, and F. *J. Virol*. (1998). 10.1128/jvi.72.10.7909-7915.1998 PMC110119 9733828

[98] Yamaguchi T (2007). Role of MyD88 and TLR9 in the innate immune response elicited by serotype 5 adenoviral vectors. Hum. Gene Ther..

[99] Appledorn DM (2008). Adenovirus vector-induced innate inflammatory mediators, MAPK signaling, as well as adaptive immune responses are dependent upon both TLR2 and TLR9 in vivo. J. Immunol..

[100] Quinn KM (2015). Antigen expression determines adenoviral vaccine potency independent of IFN and STING signaling. J. Clin. Invest..

[101] Rauch S, Jasny E, Schmidt KE, Petsch B (2018). New vaccine technologies to combat outbreak situations. Front. Immunol..

[102] Pardi N, Hogan MJ, Porter FW, Weissman D (2018). mRNA vaccines—a new era in vaccinology. Nat. Rev. Drug Discov..

[103] Iavarone C, O’Hagan DT, Yu D, Delahaye NF, Ulmer JB (2017). Mechanism of action of mRNA-based vaccines. Expert Rev. Vaccines.

[104] Simon ID, Publicover J, Rose JK (2007). Replication and propagation of attenuated vesicular stomatitis virus vectors in vivo: vector spread correlates with induction of immune responses and persistence of genomic RNA. J. Virol..

[105] Pulendran B, Ahmed R (2011). Immunological mechanisms of vaccination. Nat. Immunol..

[106] Luker KE, Hutchens M, Schultz T, Pekosz A, Luker GD (2005). Bioluminescence imaging of vaccinia virus: effects of interferon on viral replication and spread. Virology.

[107] Lin WH, Kouyos RD, Adams RJ, Grenfell BT, Griffin DE (2012). Prolonged persistence of measles virus RNA is characteristic of primary infection dynamics. Proc. Natl Acad. Sci. USA.

[108] Bachmann MF (1996). Induction of long-lived germinal centers associated with persisting antigen after viral infection. J. Exp. Med..

[109] de Veer M, Kemp J, Chatelier J, Elhay MJ, Meeusen EN (2010). The kinetics of soluble and particulate antigen trafficking in the afferent lymph, and its modulation by aluminum-based adjuvant. Vaccine.

[110] Kool M, Fierens K, Lambrecht BN (2012). Alum adjuvant: some of the tricks of the oldest adjuvant. J. Med. Microbiol..

[111] Hem, S. L. & Hogensch, H. *Relationship Between Physical and Chemical Properties of Aluminum-containing Adjuvants and Immunopotentiation* (Future Drugs Ltd., 2007). 10.1586/14760584.6.5.685 17931150

[112] Tam HH (2016). Sustained antigen availability during germinal center initiation enhances antibody responses to vaccination. Proc. Natl Acad. Sci. USA.

[113] Moyer TJ, Zmolek AC, Irvine DJ (2016). Beyond antigens and adjuvants: formulating future vaccines. J. Clin. Invest..

[114] Cirelli KM, Crotty S (2017). Germinal center enhancement by extended antigen availability. Curr. Opin. Immunol..

[115] Zinkernagel RM (2000). Localization dose and time of antigens determine immune reactivity. Semin. Immunol..

[116] Blair DA (2011). Duration of antigen availability influences the expansion and memory differentiation of T cells. J. Immunol..

[117] Shaulov A, Murali-Krishna K (2008). CD8 T cell expansion and memory differentiation are facilitated by simultaneous and sustained exposure to antigenic and inflammatory milieu. J. Immunol..

[118] Johansen P (2008). Antigen kinetics determines immune reactivity. PNAS.

[119] Benson, R. A. et al. Antigen presentation kinetics control T cell/dendritic cell interactions and follicular helper T cell generation in vivo. *Elife* **4**, 10.7554/eLife.06994 (2015). 10.7554/eLife.06994 PMC4558563 26258879

[120] Baumjohann D (2013). Persistent antigen and germinal center B cells sustain T follicular helper cell responses and phenotype. Immunity.

[121] Gupta, R. K., Chang, A. C., Griffin, P., Rivera, R. & Siber, G. R. In vivo distribution of radioactivity in mice after injection of biodegradable polymer microspheres containing ^14^C-labeled tetanus toxoid. *Vaccine* (1996). 10.1016/s0264-410x(96)00073-4 8994315

[122] Moon JJ (2012). Enhancing humoral responses to a malaria antigen with nanoparticle vaccines that expand Tfh cells and promote germinal center induction. Proc. Natl Acad. Sci. USA.

[123] Pauthner M (2017). Elicitation of robust tier 2 neutralizing antibody responses in nonhuman primates by HIV envelope trimer immunization using optimized approaches. Immunity.

[124] Cirelli KM (2019). Slow delivery immunization enhances HIV neutralizing antibody and germinal center responses via modulation of immunodominance. Cell.

[125] Brem, H. et al. Placebo-controlled trial of safety and efficacy of intraoperative controlled delivery by biodegradable polymers of chemotherapy for recurrent gliomas. *Lancet* (1995). 10.1016/s0140-6736(95)90755-6 7723496

[126] Bobo D, Robinson KJ, Islam J, Thurecht KJ, Corrie SR (2016). Nanoparticle-based medicines: a review of FDA-approved materials and clinical trials to date. Pharm. Res..

[127] Cleland, J. L. Single-administration vaccines: controlledrelease technology to mimic repeated immunizations. *Trends Biotechnol.* (1999). 10.1016/s0167-7799(98)01272-4 10098275

[128] Tzeng SY (2016). Thermostabilization of inactivated polio vaccine in PLGA-based microspheres for pulsatile release. J. Control Release.

[129] Malyala P, O’Hagan DT, Singh M (2009). Enhancing the therapeutic efficacy of CpG oligonucleotides using biodegradable microparticles. Adv. Drug Deliv. Rev..

[130] O’Hagan DT, Valiante NM (2003). Recent advances in the discovery and delivery of vaccine adjuvants. Nat. Rev. Drug Discov..

[131] Tanaka M (2019). Development of a simple new flow cytometric antibody-dependent cellular cytotoxicity (ADCC) assay with excellent sensitivity. J. Immunol. Methods.

[132] Guarecuco R (2018). Immunogenicity of pulsatile-release PLGA microspheres for single-injection vaccination. Vaccine.

[133] Katare YK, Panda AK (2006). Influences of excipients on in vitro release and in vivo performance of tetanus toxoid loaded polymer particles. Eur. J. Pharm. Sci..

[134] Jaganathan KS (2005). Development of a single dose tetanus toxoid formulation based on polymeric microspheres: a comparative study of poly(d,l-lactic-co-glycolic acid) versus chitosan microspheres. Int. J. Pharm..

[135] Jaganathan KS, Singh P, Prabakaran D, Mishra V, Vyas SP (2004). Development of a single-dose stabilized poly(d,l-lactic-co-glycolic acid) microspheres-based vaccine against hepatitis B. J. Pharm. Pharm..

[136] DeMuth PC, Min Y, Irvine DJ, Hammond PT (2014). Implantable silk composite microneedles for programmable vaccine release kinetics and enhanced immunogenicity in transcutaneous immunization. Adv. Health Mater..

[137] Kempe S, Mader K (2012). In situ forming implants—an attractive formulation principle for parenteral depot formulations. J. Control Release.

[138] Finnie KS (2008). Biodegradability of sol–gel silica microparticles for drug delivery. J. Sol–Gel Sci. Technol..

[139] Abbaraju PL (2018). Asymmetric mesoporous silica nanoparticles as potent and safe immunoadjuvants provoke high immune responses. Chem. Commun..

[140] Megumu Higaki, Y. A. et al. Collagen minipellet as a controlled release delivery system for tetanus and diphtheria toxoid. *Vaccine* (2001). 10.1016/s0264-410x(01)00039-1 11312003

[141] Kabashima K, Honda T, Ginhoux F, Egawa G (2019). The immunological anatomy of the skin. Nat. Rev. Immunol..

[142] Kim YC, Park JH, Prausnitz MR (2012). Microneedles for drug and vaccine delivery. Adv. Drug Deliv. Rev..

[143] Tsioris K (2012). Fabrication of silk microneedles for controlled-release drug delivery. Adv. Funct. Mater..

[144] Ali OA, Huebsch N, Cao L, Dranoff G, Mooney DJ (2009). Infection-mimicking materials to program dendritic cells in situ. Nat. Mater..

[145] Sullivan SP (2010). Dissolving polymer microneedle patches for influenza vaccination. Nat. Med..

[146] Kommareddy S (2012). Dissolvable microneedle patches for the delivery of cell-culture-derived influenza vaccine antigens. J. Pharm. Sci..

[147] Ferraro B (2011). Clinical applications of DNA vaccines: current progress. Clin. Infect. Dis..

[148] Li L, Petrovsky N (2016). Molecular mechanisms for enhanced DNA vaccine immunogenicity. Expert Rev. Vaccines.

[149] Marston, M. et al. Therapy by intramuscular injection of plasmid DNA: studies on firefly luciferase gene expression in mice. *Hum. Gene Therapy* (1993). 10.1089/hum.1993.4.4-419 8399489

[150] Kinnear E, Caproni LJ, Tregoning JS (2015). A comparison of red fluorescent proteins to model DNA vaccine expression by whole animal in vivo imaging. PLoS ONE.

[151] Geiben-Lynn R, Frimpong-Boateng K, Letvin NL (2011). Modulation of plasmid DNA vaccine antigen clearance by caspase 12 RNA interference potentiates vaccination. Clin. Vaccin. Immunol..

[152] Geiben-Lynn R, Greenland JR, Frimpong-Boateng K, Letvin NL (2008). Kinetics of recombinant adenovirus type 5, vaccinia virus, modified vaccinia ankara virus, and DNA antigen expression in vivo and the induction of memory T-lymphocyte responses. Clin. Vaccin. Immunol..

[153] Petkov SP (2013). Evaluation of immunogen delivery by DNA immunization using non-invasive bioluminescence imaging. Hum. Vaccin Immunother..

[154] Petkov S (2018). DNA immunization site determines the level of gene expression and the magnitude, but not the type of the induced immune response. PLoS ONE.

[155] Roos AK (2009). Skin electroporation: effects on transgene expression, DNA persistence and local tissue environment. PLoS ONE.

[156] Pardi N (2017). Zika virus protection by a single low-dose nucleoside-modified mRNA vaccination. Nature.

[157] Bahl K (2017). Preclinical and clinical demonstration of immunogenicity by mRNA vaccines against H10N8 and H7N9 influenza viruses. Mol. Ther..

[158] Hassett KJ (2019). Optimization of lipid nanoparticles for intramuscular administration of mRNA vaccines. Mol. Ther. Nucleic Acids.

[159] Pepini T (2017). Induction of an IFN-mediated antiviral response by a self-amplifying RNA vaccine: implications for vaccine design. J. Immunol..

[160] Geall AJ (2012). Nonviral delivery of self-amplifying RNA vaccines. PNAS.

[161] Zhang C, Maruggi G, Shan H, Li J (2019). Advances in mRNA vaccines for infectious diseases. Front Immunol..

[162] Yang TC (2007). On the role of CD4+ T cells in the CD8+ T-cell response elicited by recombinant adenovirus vaccines. Mol. Ther..

[163] Yang TC (2005). The CD8+ T cell population elicited by recombinant adenovirus displays a novel partially exhausted phenotype associated with prolonged antigen presentation that nonetheless provides long-term immunity. J. Immunol..

[164] Casimiro DR (2003). Comparative immunogenicity in Rhesus monkeys of DNA plasmid, recombinant vaccinia virus, and replication-defective adenovirus vectors expressing a human immunodeficiency virus type 1 gag gene. J. Virol..

[165] Buchbinder SP (2008). Efficacy assessment of a cell-mediated immunity HIV-1 vaccine (the Step Study): a double-blind, randomised, placebo-controlled, test-of-concept trial. Lancet.

[166] Ye B (2015). T-cell exhaustion in chronic hepatitis B infection: current knowledge and clinical significance. Cell Death Dis..

[167] Watanabe T, Bertoletti A, Tanoto TA (2010). PD-1/PD-L1 pathway and T-cell exhaustion in chronic hepatitis virus infection. J. Viral Hepat..

[168] Henao-Restrepo AM (2017). Efficacy and eff ectiveness of an rVSV-vectored vaccine in preventing Ebola virus disease: fi nal results from the Guinea ring vaccination, open-label, cluster-randomised trial (Ebola Ça Suffit!). Lancet.

[169] Hansen SG (2013). Cytomegalovirus vectors violate CD8+ T cell epitope recognition paradigms. Science.

[170] Adam VS (2014). Adeno-associated virus 9-mediated airway expression of antibody protects old and immunodeficient mice against influenza virus. Clin. Vaccin. Immunol..

[171] Balazs AB (2011). Antibody-based protection against HIV infection by vectored immunoprophylaxis. Nature.

[172] Wilson MPLAJM (2006). Adeno-associated virus serotype 9 vectors transduce murine alveolar and nasal epithelia and can be readministered. PNAS.

[173] Limberis, M. P. et al. Intranasal antibody gene transfer in mice and ferrets elicits broad protection against pandemic influenza. *Sci. Transl. Med.* (2013). 10.1126/scitranslmed.3006299 PMC4596530 23720583

[174] Lauer, K. B., Borrow, R. & Blanchard, T. J. Multivalent and multipathogen viral vector vaccines. *Clin. Vaccine Immunol.* **24**, 10.1128/CVI.00298-16 (2017). 10.1128/CVI.00298-16 PMC5216423 27535837

[175] Mueller, S. N. & Ahmed, R. High antigen levels are the cause of T cell exhaustion during chronic viral infection. *PNAS* (2009). 10.1073/pnas.0809818106 PMC2688997 19433785

[176] Han S, Asoyan A, Rabenstein H, Nakano N, Obst R (2010). Role of antigen persistence and dose for CD4+ T-cell exhaustion and recovery. Proc. Natl Acad. Sci. USA.

[177] Wherry EJ, Kurachi M (2015). Molecular and cellular insights into T cell exhaustion. Nat. Rev. Immunol..

[178] Hutchison S (2012). Antigen depot is not required for alum adjuvanticity. FASEB J..

[179] Moyer TJ (2020). Engineered immunogen binding to alum adjuvant enhances humoral immunity. Nat. Med..

[180] Dupuis, M., McDonald, D. M. & Ott, G. Distribution of adjuvant MF59 and antigen gD2 after intramuscular injection in mice. *Vaccine* (2000). 10.1016/s0264-410x(99)00263-7 10519932

[181] Pedersen GK, Andersen P, Christensen D (2018). Immunocorrelates of CAF family adjuvants. Semin. Immunol..

[182] Kamath AT (2012). Synchronization of dendritic cell activation and antigen exposure is required for the induction of Th1/Th17 responses. J. Immunol..

[183] Schmidt ST (2016). The administration route is decisive for the ability of the vaccine adjuvant CAF09 to induce antigen-specific CD8(+) T-cell responses: the immunological consequences of the biodistribution profile. J. Control Release.

[184] Lingnau, K., Riedl, K. & von Gabain, A. *C31® and IC30, Novel Types of Vaccine Adjuvant Based on Peptide Delivery Systems* (Future Drugs Ltd, 2007). 10.1586/14760584.6.5.741 17931154

[185] Schellack C (2006). IC31, a novel adjuvant signaling via TLR9, induces potent cellular and humoral immune responses. Vaccine.

[186] Liang F (2015). Dissociation of skeletal muscle for flow cytometric characterization of immune cells in macaques. J. Immunol. Methods.

[187] Liang F, Lore K (2016). Local innate immune responses in the vaccine adjuvant-injected muscle. Clin. Transl. Immunol..

[188] Vono M (2013). The adjuvant MF59 induces ATP release from muscle that potentiates response to vaccination. Proc. Natl Acad. Sci. USA.

[189] Didierlaurent AM (2014). Enhancement of adaptive immunity by the human vaccine adjuvant AS01 depends on activated dendritic cells. J. Immunol..

[190] Lu F, Hogenesch H (2013). Kinetics of the inflammatory response following intramuscular injection of aluminum adjuvant. Vaccine.

[191] Calabro S (2011). Vaccine adjuvants alum and MF59 induce rapid recruitment of neutrophils and monocytes that participate in antigen transport to draining lymph nodes. Vaccine.

[192] Zaric, M. et al. Skin Dendritic cell targeting via microneedle arrays laden with antigen-encapsulated poly‑d,l‑lactideco-glycolide nanoparticles induces efficient antitumor and antiviral immune responses. *ACS Nano* (2013). 10.1021/nn304235j PMC3936823 23373658

[193] Singh A, Suri S, Roy K (2009). In-situ crosslinking hydrogels for combinatorial delivery of chemokines and siRNA-DNA carrying microparticles to dendritic cells. Biomaterials.

[194] Baleeiro RB (2013). Topical vaccination with functionalized particles targeting dendritic cells. J. Invest. Dermatol..

[195] Reddy ST (2007). Exploiting lymphatic transport and complement activation in nanoparticle vaccines. Nat. Biotechnol..

[196] Swartz, M. A. The physiology of the lymphatic system. *Adv. Drug Deliv. Rev.* (2001). 10.1016/s0169-409x(01)00150-8 11489331

[197] Rantakari P (2015). The endothelial protein PLVAP in lymphatics controls the entry of lymphocytes and antigens into lymph nodes. Nat. Immunol..

[198] Jalkanen S, Salmi M (2020). Lymphatic endothelial cells of the lymph node. Nat. Rev. Immunol..

[199] Sixt M (2005). The conduit system transports soluble antigens from the afferent lymph to resident dendritic cells in the T cell area of the lymph node. Immunity.

[200] Gretz JE, Norbury CC, Anderson AO, Proudfoot AEI, Shaw SLymph-borne (2000). Chemokines and other low molecular weight molecules reach high endothelial venules via specialized conduits while a functional barrier limits access to the lymphocyte microenvironments in lymph node cortex. J. Exp. Med..

[201] Nuhn L (2016). pH-degradable imidazoquinoline-ligated nanogels for lymph node-focused immune activation. Proc. Natl Acad. Sci. USA.

[202] Roozendaal R (2009). Conduits mediate transport of low-molecular-weight antigen to lymph node follicles. Immunity.

[203] Catron DM, Pape KA, Fife BT, van Rooijen N, Jenkins MK (2010). A protease-dependent mechanism for initiating T-dependent B cell responses to large particulate antigens. J. Immunol..

[204] Gonzalez SF (2010). Complement-dependent transport of antigen into B cell follicles. J. Immunol..

[205] Park, C., Arthos, J., Cicala, C. & Kehrl, J. H. The HIV-1 envelope protein gp120 is captured and displayed for B cell recognition by SIGN-R1(+) lymph node macrophages. *Elife* **4**, 10.7554/eLife.06467 (2015). 10.7554/eLife.06467 PMC4574315 26258881

[206] Manolova V (2008). Nanoparticles target distinct dendritic cell populations according to their size. Eur. J. Immunol..

[207] van Rooijen, N. & Hendrikx, E. Liposomes for specific depletion of macrophages from organs and tissues. *Methods Mol. Biol*. (2010). 10.1007/978-1-60327-360-2_13 20072882

[208] van Rooijen, N. & van Kesteren-Hendrikx, E. Clodronate liposomes: perspectives in research and therapeutics. *J. Liposome Res*. (2002). 10.1081/lpr-120004780 12604042

[209] Kaplan DH (2010). In vivo function of Langerhans cells and dermal dendritic cells. Trends Immunol..

[210] Liang F (2017). Vaccine priming is restricted to draining lymph nodes and controlled by adjuvant-mediated antigen uptake. Sci. Transl. Med..

[211] Azzi J (2016). Targeted delivery of immunomodulators to lymph nodes. Cell Rep..

[212] Jackson LA (2020). An mRNA vaccine against SARS-CoV-2—preliminary report. N. Engl. J. Med..

[213] Folegatti PM (2020). Safety and immunogenicity of the ChAdOx1 nCoV-19 vaccine against SARS-CoV-2: a preliminary report of a phase 1/2, single-blind, randomised controlled trial. Lancet.

[214] Zhu F-C (2020). Immunogenicity and safety of a recombinant adenovirus type-5-vectored COVID-19 vaccine in healthy adults aged 18 years or older: a randomised, double-blind, placebo-controlled, phase 2 trial. Lancet..

[215] Mercado NB (2020). Single-shot Ad26 vaccine protects against SARS-CoV-2 in rhesus macaques. Nature..

